# Are serum-free and xeno-free culture conditions ideal for large scale clinical grade expansion of Wharton’s jelly derived mesenchymal stem cells? A comparative study

**DOI:** 10.1186/scrt477

**Published:** 2014-07-28

**Authors:** Priyanka Swamynathan, Parvathy Venugopal, Suresh Kannan, Charan Thej, Udaykumar Kolkundar, Swaroop Bhagwat, Malancha Ta, Anish Sen Majumdar, Sudha Balasubramanian

**Affiliations:** Stempeutics Research Pvt Ltd, Akshay Tech Park #72 & 73, 2nd Floor, EPIP Zone, Phase 1, Whitefield, Bangalore 560066 India; IISER, Kolkata, India

## Abstract

**Introduction:**

Mesenchymal stromal/stem cells (MSCs) for clinical use have largely been isolated from the bone marrow, although isolation of these cells from many different adult and fetal tissues has been reported as well. One such source of MSCs is the Whartons Jelly (WJ) of the umbilical cord, as it provides an inexhaustible source of stem cells for potential therapeutic use. Isolation of MSCs from the umbilical cord also presents little, if any, ethical concerns, and the process of obtaining the cord tissue is relatively simple with appropriate consent from the donor. However, a great majority of studies rely on the use of bovine serum containing medium for isolation and expansion of these cells, and porcine derived trypsin for dissociating the cells during passages, which may pose potential risks for using these cells in clinical applications. It is therefore of high priority to develop a robust production process by optimizing culture variables to efficiently and consistently generate MSCs that retain desired regenerative and differentiation properties while minimizing risk of disease transmission.

**Methods:**

We have established a complete xeno-free, serum-free culture condition for isolation, expansion and characterization of WJ-MSCs, to eliminate the use of animal components right from initiation of explant culture to clinical scale expansion and cryopreservation. Growth kinetics, *in vitro* differentiation capacities, immunosuppressive potential and immunophenotypic characterization of the cells expanded in serum-free media have been compared against those cultured under standard fetal bovine serum (FBS) containing medium. We have also compared the colony-forming frequency and genomic stability of the large scale expanded cells. Secretome analysis was performed to compare the angiogenic cytokines and functional angiogenic potency was proved by Matrigel assays.

**Results:**

Results presented in this report identify one such serum-free, xeno-free medium for WJ expansion. Cells cultured in serum-free, xeno-free medium exhibit superior growth kinetics and functional angiogenesis, alongside other MSC characteristics.

**Conclusions:**

We report here that WJ-MSCs cultured and expanded in Mesencult XF, SF Medium retain all necessary characteristics attributed to MSC for potential therapeutic use.

**Electronic supplementary material:**

The online version of this article (doi:10.1186/scrt477) contains supplementary material, which is available to authorized users.

## Introduction

Mesenchymal stem cells (MSCs), also known as multipotent stromal cells or mesenchymal progenitor cells (MPCs), have gained attention in regenerative medicine and tissue engineering applications ultimately leading to potential tissue repair due to their regenerative capability, multilineage differentiation potential and immunomodulatory activity [1–4]. MSCs were originally isolated and characterized from bone marrow (BM) [5], but subsequently have been derived from almost all postnatal tissues, such as adipose, dental pulp, umbilical cord and cord blood, amniotic fluid, limbal tissue and so on [6–8]. Clinical studies employing human MSCs from different sources have been initiated for treatment of several diseases, such as graft-versus host disease (GvHD), cartilage regeneration, myocardial infarction, diabetes, peripheral arterial disease and so on (http://clinicaltrials.gov/).

Although BM is the traditional and the most well characterized source of human MSCs, it has certain limitations, such as subjecting the donors to painful isolation, decline in MSC precursor frequency with age, reduced proliferation capacity and non-optimal differentiation potential of these cells. When compared against other tissues, the umbilical cord appears to provide an inexhaustible source of stem cells for therapy and use of this tissue would not involve invasive procedures or ethical concerns. MSCs have been isolated from different compartments of the umbilical cord; Wharton’s Jelly (WJ) is the embryonic mucous connective tissue lying between the amniotic epithelium and the umbilical vessels and is a rich source of MSCs [9–11]. WJ-MSCs share some properties unique to fetal-derived MSCs, such as faster proliferation and greater *ex vivo* expansion capabilities, compared to adult MSCs [12].

Most of the MSC expansion procedures involve the use of bovine serum containing medium for culturing the cells and porcine derived trypsin for dissociating the cells and the use of these two ingredients poses a potential risk of transmitting unknown viruses, mycoplasma, prions or unidentified zoonotic agents. Moreover, the presence of a high quantity of xenogeneic proteins can cause concerns related to immune reactions in human patients [13]. Also, a high degree of batch-to-batch variation could cause inconsistency in generating quality-controlled cells, thus making standardization of the production process difficult. Although the use of animal serum for cell expansion is not prohibited, the development of serum substitutes and xeno-free and serum-free medium for MSC has become a high priority for these reasons. In general, the current protocols for clinical grade production involve isolation of a small fraction of primary MSCs from the selected tissue source and expanding them through multiple passages, in order to generate clinically useful numbers of cells. Subsequently, these cells are used for the intended clinical applications [14]. Several cell culture variables, such as medium formulation (basal media and supplements), cell surface attachment substrate and cell seeding density at various passages, are critical when developing a potential cell therapy based product. Keeping these points in mind, we have compared two commercially available xeno-free and serum-free media, StemPro XF, SFM (Invitrogen, Carlsbad, CA, USA) and MesenCult XF, SFM (Stem Cell Technologies, Vancouver, British Columbia, Canada), to establish and expand WJ-MSCs. While an earlier study by Corotchi *et al*., [15] demonstrated the isolation and culture of WJ-MSCs in MesenCult medium and their endothelial differentiation propensity, we have optimized the culture conditions of WJ-MSCs in Mesencult for large scale expansion and performed extensive characterization based on the requirements of using these cells in clinical trials. In addition, we have also eliminated the use of animal components right from the isolation step to the clinical scale expansion process. These WJ-MSCs have been characterized with respect to their growth kinetics, *in vitro* differentiation capacity, immunosuppressive potential and immunophenotypic analysis. Our study presents the superiority of the cells cultured in xeno-free, serum-free conditions when compared against those grown and expanded under conventional fetal bovine serum (FBS) culture conditions. These results suggest that the expanded WJ-MSCs met all necessary regulatory criteria in order to evaluate their therapeutic potential in pre-clinical and clinical studies.

## Methods

### MSC isolation and cell culture

Fresh human umbilical cords (n = 10) were obtained after full-term births (cesarean section or normal vaginal delivery) with written informed consent using the guidelines approved by the Institutional Committee for Stem Cell Research and Therapy (ICSCRT) and the Institutional Ethics Committee (IEC) at the Manipal Hospital, Bangalore, India. The study was also approved by both the committees.

Briefly, after rinsing with normal saline (0.9% w/v sodium chloride), the cords were given a quick rinse with 70% isopropyl alcohol (IPA), followed by three washes with sterile PBS. The umbilical cord vessels were then removed manually from cord segments, and the exposed mesenchymal tissue was cut into very small pieces or explants, approximately 1 to 2 mm, before placing them in a tissue culture dish. The explants were cultured in:

1. DMEM KO (Cat.no. 10829018, Invitrogen, Carlsbad, CA, USA) and 2 mM L-glutamine (Cat.no., 25030, Invitrogen), supplemented with 10% FBS Cat.no.SH30084.03, Lot no.GRH0054 (Hyclone, Waltham, MA, USA);
2. MesenCult XF Medium (Cat.no. 05429, Stem Cell Technologies, Vancouver, British Columbia, Canada), supplemented with 200 mM L-glutamine (Cat.no. 25030, Invitrogen); prior to plating, culture dishes were coated with MesenCult XF Attachment Substrate (Cat.no. 05424, Stem Cell Technologies);
3. StemPro XF, SF media (Cat.no. A10675, Invitrogen), supplemented with 200 mM L-glutamine (Cat.no. 25030, Invitrogen); prior to plating, culture dishes were coated with CELLstart CTS Attachment Substrate (Cat.no. A1014201, Invitrogen) according to the manufacturers’ protocols.

For subculturing, 0.05% trypsin-ethylenediaminetetraacetic acid (EDTA) (Gibco, Life Technologies - Carlsbad, CA, USA, cat.no. 25300-054) was used for the FBS cultures, while TryplE Select 10X (Gibco, cat.no. A12177-01) was used for the XF, SFM cultures. WJ-MSCs were expanded in culture up to passage 5 to study their growth kinetics. Representative pictures were captured at 10 X to compare morphology of the cells. Mean population doubling, cumulative population doubling and total cell yield were calculated and compared between the two culture conditions.

### Colony forming unit-fibroblast assay

To test the colony forming capacity of the MSCs, the cells were plated at a density of 100 cells per 35 mm dish. The cells were incubated for eight days, at which point they were fixed and stained with 0.1% toluidene blue in 1% paraformaldehyde (all reagents from Sigma-Aldrich, St. Louis, MO, USA), to visualize the colonies. Stained colonies were manually counted. The assay for each sample was carried out in triplicate.

### Immunophenotyping

In order to screen for the presence of MSC positive and negative markers and to confirm the absence of co-stimulatory molecules, cells at Passage 3 and Passage 5, cultured in all three conditions were subjected to flow cytometry analysis. The samples were incubated with the below mentioned antibodies for half an hour at room temperature, following which the samples were analyzed using the FACS DIVA and WinMDI 2.9 software. The expression levels of CD 73, CD 105, CD 44, CD 166, CD 90, HLA ABC, CD 34, CD 45, HLA DR, CD 40, CD 80 and CD 86 were analyzed. The details and concentrations of the antibodies used are given in Table 1.

**Table 1:** **Antibodies used for flow cytometry analysis**

Antibody	Fluorochrome	Catalogue number	Stock concentraton	Antibody dilution	Isotype dilution
CD73	PE	550257, BD	12.5 µg/ml	NEAT	1:16 ISO PE
CD44	PE	550989, BD	12.5 µg/ml	NEAT	1:16 ISO PE
CD166	PE	559263, BD	12.5 µg/ml	NEAT	1:16 ISO PE
CD90	PE	555596, BD	0.2 mg/ml	NEAT	NEAT ISO PE
CD105	FITC	FAB10971F, R&D	50 µg/ml	NEAT	1:10 ISO FITC
CD200	PE	552475, BD	0.25 µg/20 ml	NEAT	NEAT ISO PE
HLA ABC	APC	555555, BD	3 µg/ml	NEAT	1:4 ISO APC
CD34	PE	550761, BD	12.5 µg/ml	NEAT	1:16 ISO PE
CD271	PE	557196, BD	0.125 µg/20 ml	NEAT	NEAT ISO PE
CD80	PE	340294, BD	0.0032 mg/ml	NEAT	1:62.5 ISO PE
CD45	FITC	555482, BD	6.25 µg/ml	NEAT	1:80 ISO FITC
HLA DR	FITC	347663, BD	25 µg/ml	NEAT	1:2 ISO FITC

APC, allophycocyanin; FITC, fluorescein isothiocyanate; PE, phycoerythrin.

### RT-PCR analysis

Total cellular RNA was isolated using an RNeasy mini kit (Qiagen, Venlo, Limburg, Netherlands). The RNA samples were treated with DNA-free *DNase I* (Ambion, Life Technologies - Carlsbad, CA, USA), according to the manufacturer’s instructions and reverse-transcribed into cDNA using a high capacity cDNA reverse transcription kit (Applied Biosystems, Life Technologies - Carlsbad, CA, USA) according to the manufacturer’s instructions. Transcripts were amplified using gene specific primers, for which the primer sequences are provided in Table 2. A reverse transcriptase negative blank of each sample and a no template blank served as negative controls. Gene expression was normalized to the housekeeping gene beta actin, used as an internal standard.

**Table 2:** **List of primers used for RT-PCR analysis**

Markers	Forward primer	Reverse primer	Tm	Product length
β-actin	CATGTACGTTGCTATCCAGGC	CTCCTTAATGTCACGCACGAT	60	250 bp
P21	GAGGCCGGGATGAGTTGGGAGGAG	CAGCCGGCGTTTGGAGTGGTAGAA	63	220 bp
P53	TAACAGTTCCTGCATGGGCGGC	AGGACAGGCACAAACACGCACC	60	121 bp
P16	TTATTTGAGCTTTGGTTCTG	CCGGCTTTCGTAGTTTTCAT	51	354 bp
XRCC4	AAGATGTCTCATTCAGACTTG	CCGCTTATAAAGATCAGTCTC	54	233 bp
PTN	GGAGCTGAGTGCAAGCAAAC	GAGGTTTGGGCTTGGTCAGT	59	274 bp
MCM3	TCGAGGTAACCTTTGGTGGC	TGTTCAGAAGCCTCGTCGTC	55	232 bp
CDKN2B	CCCAACTCCACCAGATAGCA	GGGATTTCCGCATCCTAGCA	57	198 bp
ERCC3	CCAGGAAGCGGCACTATGAGG	GGTCGTCCTTCAGCGGCATTT	61	171 bp
Cmyc	AAGACTCCAGCGCCTTCTCTC	GTTTTCCAACTCCGGGATCTG	62	526 bp
VEGF-A	GCAGAATCATCACGAAGTGG	GCATGGTGATGTTGGACTCC	60	234 bp
TGF-β	CAGATCCTGTCCAAGCTG	TCGGAGCTCTGATGGTGTT	56	270 bp
Ang-1	GCTTACCAGATTCACACTGTTCC	TTGCTACCTTGCCAACAACAACTG	60	612 bp
IL-6	CACAGACAGCCACTCACCTC	TTTTCTGCCAGTGCCTCTTT	60	137 bp
HGF	ATGCATCCAAGGTCAAGGAG	TTCCATGTTCTTGTCCCACA	61	349 bp

### Tri-lineage differentiation and quatification

Tri-lineage differentiation of WJ-MSCs into adipocytes, osteocytes and chondrocytes was carried out using previously described techniques [10].

Adipogenic differentiation was initiated in confluent cultures of MSCs using complete medium supplemented with 200 mM indomethacin, 0.5 mM 3-isobutyl-1-methylxanthine, 10 mg/ml insulin and 1 mM dexamethasone (all reagents from Sigma-Aldrich). After 18 days, adipogenic differentiation was detected by staining the lipid droplets with Oil Red O (Sigma-Aldrich). For quantitation of lipid accumulation, the Oil Red O was extracted using a mixture of chloroform and methanol at a ratio of 2:1 and the absorbance at 540 nm was measured against a blank solvent using a spectrophotometer (SpectraMax M3, Molecular Devices, Sunnyvale, CA, USA). Each sample was differentiated, stained and quantified in duplicate. Osteogenic differentiation was induced in confluent cultures of MSCs using complete medium supplemented with 0.1 mM dexamethasone, 10 mM beta-glycerophosphate and 0.2 mM ascorbic acid (all reagents from Sigma-Aldrich). After 18 days, mineralization was detected by staining with Alizarin Red S (Sigma-Aldrich). Mineralization was quantified by extraction and measurement of Alizarin Red S uptake. The stained sample was treated with 800 µl 10% acetic acid for 30 minutes on a shaker at room temperature. The cell layer was then scraped, transferred into a 1.5 ml tube, heated at 85°C for 10 minutes, cooled and centrifuged at 20,000 x g for 15 minutes. The supernatant was neutralized with 200 ml 10% v/v ammonium hydroxide and the absorbance at 405 nm was measured against the blank solvent, using a plate reader (SpectraMax M3, Molecular Devices). Each sample was differentiated, stained and quantified in duplicate. Chondrogenic differentiation was brought about using the StemPro Chondrogenesis Kit (Invitrogen; cat no. A10071-01); subsequent sulfated glycosaminoglycan quantification was carried out using the Blyscan Sulfated Glycosaminoglycan Quantification Kit (Biocolor, Carrickfergus, County Antrim, UK, Product Code: B3000), after normalizing DNA.

### Enzyme-linked immunosorbent assay

Levels of angiogenic cytokines – vascular endothelial growth factor (VEGF), transforming growth factor β-1 (TGF-β-1), hepatocyte growth factor (HGF) (R&D Systems, Minneapolis, USA) and angiopoietin 1 (Ray Biotech, Norcross, GA, USA) were estimated using ELISA kits, according to the manufacturers’ instructions. The samples were assayed in duplicate. In order to negate the possibility of FBS contributing to the high level of cytokine expression, we used human-specific ELISA kits. Also, the respective complete media were used as controls.

### Angiogenesis assay

Growth factor reduced Matrigel (Cat. No. 356231, BD Biosciences, San Jose, CA, USA) was coated onto 48-well plates and allowed to solidify at 37°C for 30 minutes. Human umbilical vein endothelial cells (HUVEC, 2 × 10^4^) were suspended in the desired media condition to a total volume of 250 µl and plated onto the GFR Matrigel. An anti-hHGF polyclonal neutralizing antibody (Cat. No. ab10679, Abcam, Cambridge, England, UK) was used for the blocking study. Plates were incubated for six hours in 5% CO_2_ at 37°C. Images at a 4 X magnification were taken using an inverted phase contrast microscope (Nikon). The mean tube length and the mean number of branch points were analyzed using WimTube software (Wimasis, GmbH, Munich, Germany) from three random fields.

### Imunosuppression and immunogenicity

For stimulation assays, MSCs treated with 10 µg/ml mitomycin C (Sigma-Aldrich) for 2.5 hours were used as stimulator cells, and allogeneic peripheral blood mononuclear cells (PBMC) isolated by density gradient centrifugation were used as responder cells. MSCs were seeded in 96-well plates (Corning, NY, USA) and allowed to attach overnight. Responder PBMCs were then added to each well, at a ratio of 1:2.5 (MSCs:responder) and the cultures were maintained for five days, at which point they were pulsed with 5-bromo-2-deoxyuridine (BrdU) for the final 24 hours. Cell proliferation was measured using a fluorimetric immunoassay kit (Calbiochem, San Diego, CA, USA) for the quantification of BrdU incorporation, according to the manufacturer’s instructions. One-way mixed lymphocyte reactions (MLR) at the corresponding stimulator: responder ratio were used as positive controls for PBMC proliferation in response to alloantigen. For immunosuppression assays, mitomycin C-treated MSCs were seeded in 96-well plates and allowed to attach overnight. A one-way MLR at a ratio of 1:2.5 was then added to each well. Lymphocyte proliferation was measured at the end of five days, as described above. A one-way MLR cultured in the absence of MSCs was considered as the 100% proliferation control. Four individual donors were analyzed in each group. All treatments were performed in triplicate wells in Roswell Park Memorial Institute (RPMI) 1640 medium (Invitrogen) supplemented with 10% FBS (Hyclone), 2 mM glutamine (Invitrogen) and 0.05 mM beta-mercaptoethanol (Sigma-Aldrich).

### Senescence, *in vitro*tumorigenecity and transformation assays

#### *In vitro*tumorigenicity assay

We used a soft agar assay to assess the transformation of MSCs *in vitro*. For this, cells at passage 5 were harvested, counted using a hemocytometer and resuspended in growth media. The bottom layer of the soft agar plate was made with 0.6% agarose, poured in a six multi-well dish and allowed to settle for one hour at room temperature. The harvested cells were mixed with 0.3% agarose at a concentration of 8,000 cells/well and were overlaid above the bottom agar. The plates were pre-incubated for 30 minutes at room temperature before they were shifted to a CO_2_ incubator and were replenished with fresh media at an interval of two to three days. The formation of colonies was analyzed after 28 days and, as a positive control, MCF-7 cells were cultured simultaneously. Colonies of sizes greater than 100 microns under 10 X magnification were considered transformed.

#### Senescence assay

Cells grown in MesenCult XF SF media and DMEM KO + 10% FBS culture conditions were comparatively analyzed for the presence of senescent cells in culture, at the end of passages 3 and 5. To do so, the cells were cultured up to 60% confluence in 35 mm dishes and were fixed and stained using a Senescence β-Galactosidase Staining Kit (Cell Signaling Technology, Danvers, MA, USA, cat.no. 9860), according to the manufacturer’s instructions. After an over-night incubation with the staining solution, ten 10 X pictures per dish were taken under the microscope. Senescent cells were manually counted in each field. The assays were carried out in duplicate.

#### Annexin V staining

We looked for the presence of apoptotic cells among cultures in DMEM KO + 10% FBS and MesenCult, using the Tali Apoptosis Kit – Annexin V Alexa Flour 488 and propidium iodide (PI; cat no. 10788; Invitrogen), according to the manufacturer’s instructions. After staining the cells with Annexin V Alexa Flour 488 and PI, the samples were loaded onto Tali Cellular Analysis Slides (cat no. T10794; Invitrogen), following which 20 images were captured using the Tali Image – Based Cytometer (cat no. T10796; Invitrogen).

#### Cell cycle analysis

In order to analyze the percentage of cells in S phase at passage 6, cells grown in DMEM KO + 10% FBS and in MesenCult media were subjected to PI staining. Passage 6 cultured cells were harvested, resuspended in Dulbecco’s PBS (DPBS) (Invitrogen) and fixed using 70% ethanol. The cells were incubated for 15 minutes on ice. They were then centrifuged and resuspended in 500 µL PI solution in DPBS (50 µg/mL PI), which also contained 0.1 mg/ml RNase A and 0.05% Triton X-100. The cells were incubated for 40 minutes at 37°C, after which they were centrifuged and resuspended in DPBS for flow analysis.

#### Karyotype analysis

To eliminate the possibility of chromosomal abnormalities, karyotype analyses were carried out by making metaphase spreads of cells cultured in DMEM KO + 10% FBS and in MesenCult. The cultures were treated with colchicine, following which they were harvested, briefly treated with a hypotonic solution and fixed. The fixed cells were then dropped on to a slide, dried and stained to observe a G banding pattern.

### Statistical analysis

Data are presented as mean ± SD. The statistical significance was calculated using Student’s *t* test followed by multiple comparison tests using Graph Pad Prism. *P* <0.05 was considered statistically significant.

## Results

### Media standardization for the isolation and expansion of WJ-MSCs in xeno-free and serum-free conditions

**Figure 1:**
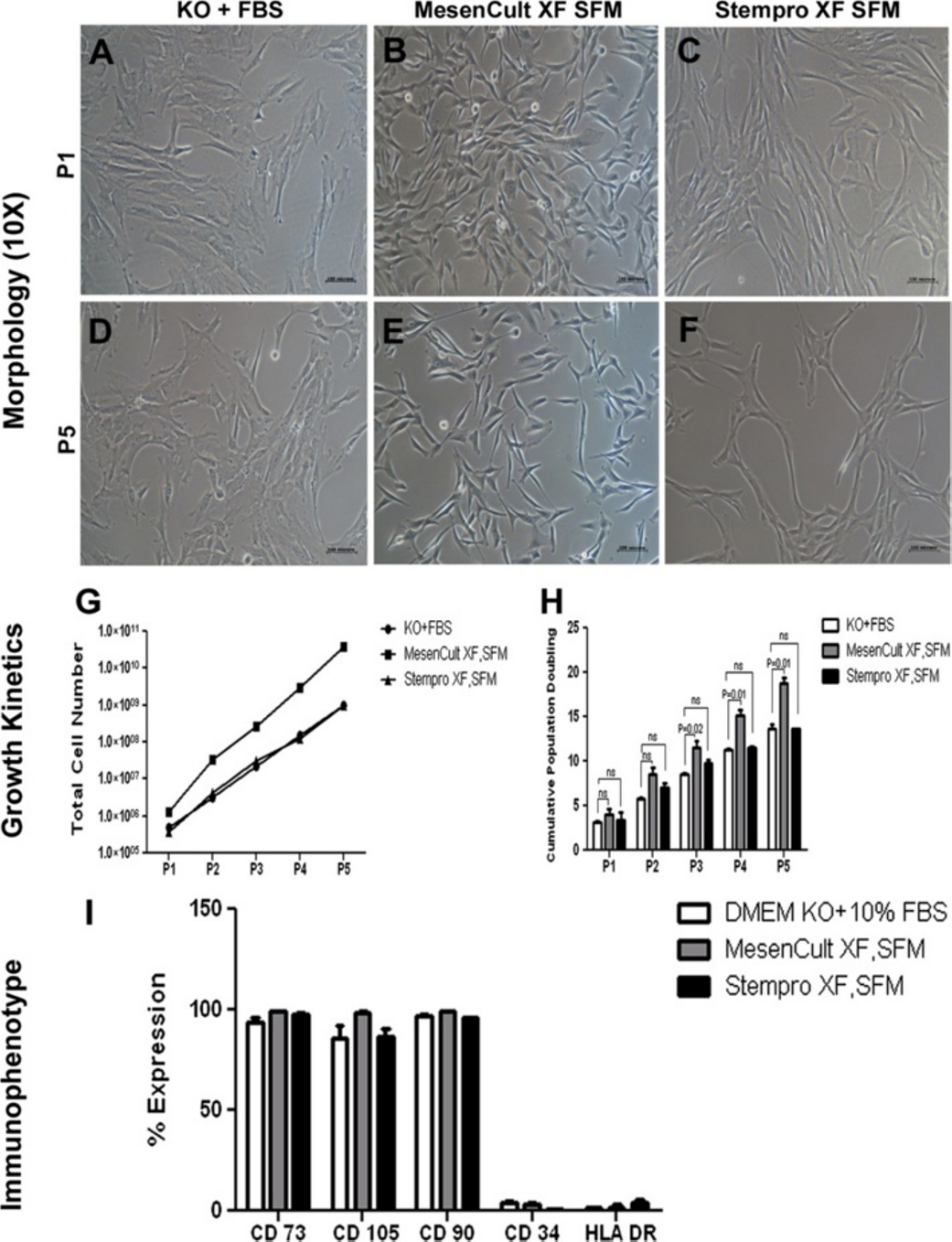
**Growth kinetics and immunophenotype. A,B,C)** Morphology pictures of WJ-MSCs grown in serum-containing and serum-free media, passage 1; **D,E,F)** Pictures of WJ- MSCs grown in serum-containing and serum-free media, passage 5; **G,H)** Total cell number and cumulative population doublings obtained upon culturing WJ-MSCs in serum-containing and serum-free media, up to passage 5; **I)** Percentage expression of MSC positive and negative makers, expressed on WJ-MSCs cultured in three different media. WJ-MSCs, Wharton’s jelly-mesenchymal stem cells.

We adapted the explant culture method for the isolation of WJ-MSCs from the umbilical cord according to the protocol mentioned in the Methods section, for two xeno-free, serum-free media from two different vendors and compared them against standard DMEM KO + 10% FBS culture conditions. The explant culture method allowed us to reproducibly isolate WJ-MSCs from the umbilical cords (n = 3) using StemPro XF, SFM and Mesencult XF, SFM. WJ-MSCs isolated from the cords were expanded in T25 or T75 flasks to passage 5 to study the growth kinetics and surface marker expression profile. We observed a significant difference in the morphology of the isolated WJ-MSCs between xeno-free conditions and FBS containing medium. In the xeno-free culture, the cells exhibited small, spindle shaped morphology whereas in the standard serum conditions, they showed a flattened fibroblast-like morphology (Figure 1A-F). We were able to successfully expand the cells from passage 0 to passage 5 for all three cords in both Mesencult and DMEM KO +10% FBS, whereas the StemPro XF SFM supported the growth of only one cord until passage 5. The remaining two WJ-MSC cultures did not propagate sufficiently beyond passage 2 and passage 4, respectively. At passage 5, total cell yield for DMEM KO + FBS, MesenCult XF,SFM and StemPro XF,SFM were 9.7E + 08 ± 8.2E + 08, 3.7E + 10 ± 3.9E + 10, 5.1E + 08 ± 6.8E + 08 and cumulative population doublings were 13.64 ± 0.87, 18.72 ± 1.18, 9.12 ± 6.26, respectively (Figure 1G,H).

*Ex vivo* expanded WJ-MSCs were immunophenotypically characterized by flow cytometry and the results are presented in Figure 1I. With regard to the expression of positive markers, such as CD73, CD105 and CD90, and negative markers, such as CD34 and HLA-DR, we did not observe a significant difference between the cells grown in the three culture conditions.

### Large scale expansion of WJ-MSCs using MesenCult XF, SF medium

Due to the inconsistent results obtained using StemPro XF, SF medium in isolating and expanding WJ-MSC in our lab, upscaling studies were performed only with Mesencult XF, SF medium (here onwards referred to as XF, SFM). Before the large scale expansion, WJ-MSCs were isolated from seven new cords in this medium, to further validate the consistency of our isolation process. WJ-MSCs isolated in Mesencult were expanded individually to passage 5, in one cell stacks, to study their growth kinetics. Morphological characteristics of these cells expanded from passage 0 to passage 5 are given in Additional file 1: Figure S1 (A-E). Mean population doubling (PD) time and cumulative population doublings (CPD) of WJ-MSCs in MesenCult medium were 28.8 ± 4.09 hours and 18.07 ± 1.21 hours, respectively. We did not observe a significant difference in the expansion capability among the individual cords except for one cord (designated as cord 7), where the average doubling time went up to 37.11 hours (Additional file 1: Figure S1). The CPD and total cell yield of the individual cords expanded in xeno-free medium from passage 0 to passage 5 are shown in Additional file 1: Figures S1:F and S1:G. The results obtained from these studies clearly suggest that the *ex vivo* growth of WJ-MSC, at least during the early passages, is highly comparable between the different cords tested.

**Figure 2:**
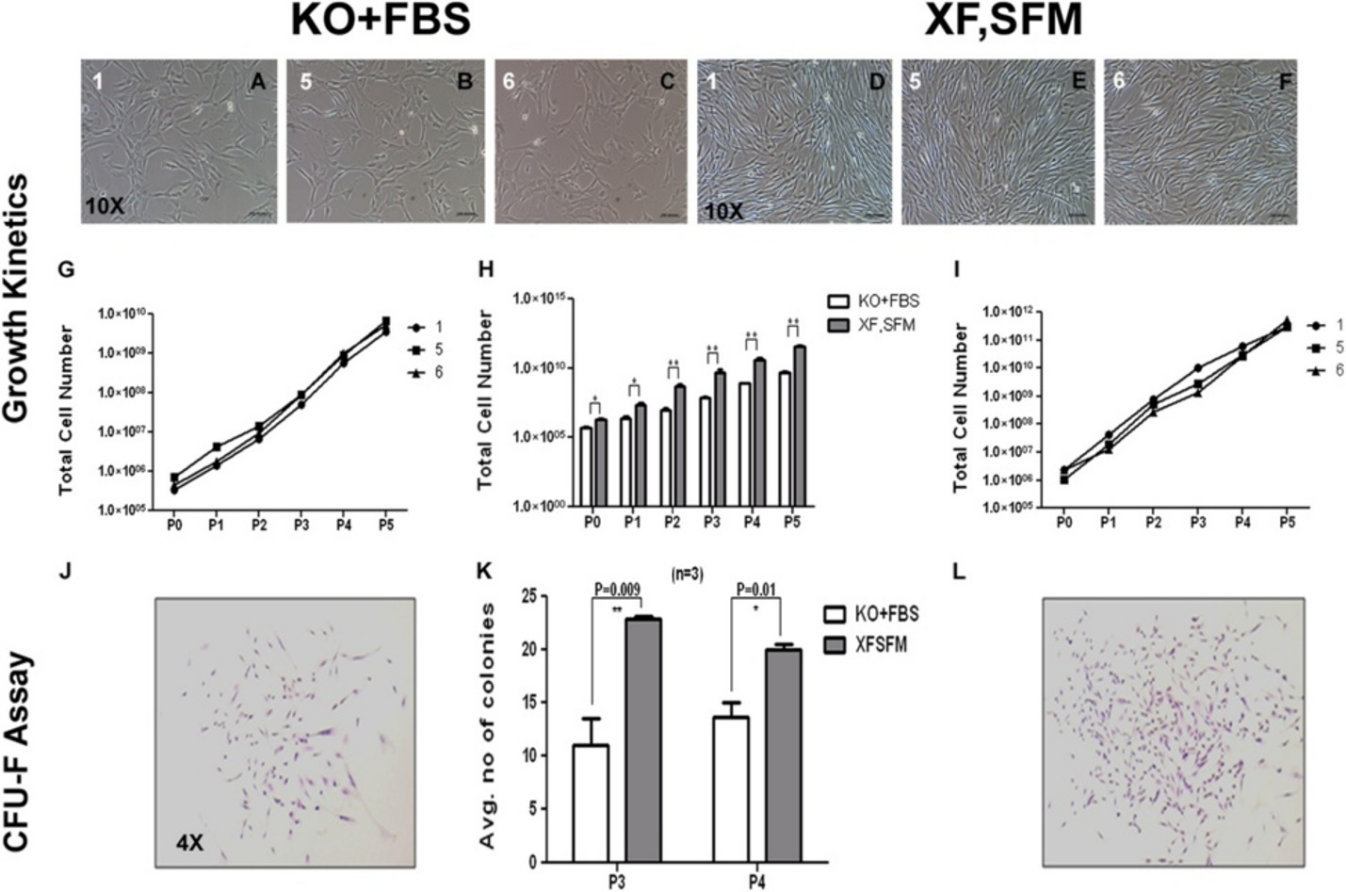
**Growth kinetics and CFU-F efficacy. A-F)** Representative morphology pictures of WJ derived MSCs cultured in DMEM KO + 10% FBS and MesenCult XF,SF media; **G,H,I)** Total cell yield obtained at passage 5 using XF,SF medium, compared against DMEM KO + 10% FBS; **K)** Average number of colonies generated per 100 cells at passage 3 and passage 4, by cells cultured in XF,SF media and DMEM KO + 10% FBS; **J,L)** Individual colonies captured at 4X magnification, for XF,SFM cultures and DMEM KO + 10% FBS cultures, respectively. FBS, fetal bovine serum; GFU-F, colony-forming unit-fibroblast; MSCs, mesenchymal stem cells; WJ, Wharton’s jelly.

Next, three individual cords were chosen for large scale expansion in XF, SFM and were compared against the same cells grown in serum containing medium. The cells were isolated and expanded until passage 2 in T 75 cm^2^ flasks, at 3,000 cells per cm^2^. At the end of passage 2, the cells were seeded in a single cell stack for large scale expansion and cultured up to passage 5. Cell morphology was observed by phase contrast microscopy and the results are shown in Figure 2A-F. We estimated the total yield of WJ-MSCs at the end of passage 5 from all the cultures. The cumulative cell yield ranged between 3.71E + 11 ± 1.2E + 11 for XF-SF medium and 5.21E + 09 ± 1.6E + 09 for DMEM KO + 10% FBS medium (Figure 2G and I). The total cell yield from the XF, SFM culture was significantly higher (*P* <0.003) than that of the cells grown in DMEM KO + 10% FBS at all the passages tested (passage 0 to passage 5) (Figure 2H). The CPDs reached a peak of 18.32 ± 0.38 at the end of passage 5 in XF, SFM as compared with 16.25 ± 0.26 in DMEM KO + 10% FBS containing medium (*P* <0.02) (Additional file 2: Figure S2: A). The mean population doubling times of large scale expanded cells were observed to be 32.13 ± 3.95 hours and 45.24 ± 4.35 hours (*P* <0.01) for serum-free and serum-containing media, respectively (Additional file 2: Figure S2: B). It is evident from these results that cells cultured and expanded in XF, SFM proliferate faster and the average yield is significantly higher than those grown in serum containing medium.

### Clonogenic potential of WJ-MSCs

The colony forming unit-fibroblast (CFU-F) assay was performed between the WJ-MSCs expanded in XF, SFM and serum containing medium for passage 3 and Passage 4 (Figure 2J, K, and L). Cells expanded in MesenCult had an average CFU-F of 23.33 ± 0.57 at passage 3 and 20 ± 1.0 at passage 4 whereas the cells expanded in the DMEM KO + 10% FBS had 11 ± 1.5 at passage 3 and 13 ± 3.08 at passage 4. The total number of colonies was significantly higher with the cells expanded in XF, SFM when compared to the cells in FBS containing medium (*P* = 0.004). Surprisingly, unlike MesenCult grown cells, we observed a difference in the number of CFU-Fs between the FBS grown cells at passage 3 and passage 4. However, this increase was not found to be significant (*P* = 0.3190).

### Immunophenotypic analysis of WJ-MSC

**Figure 3:**
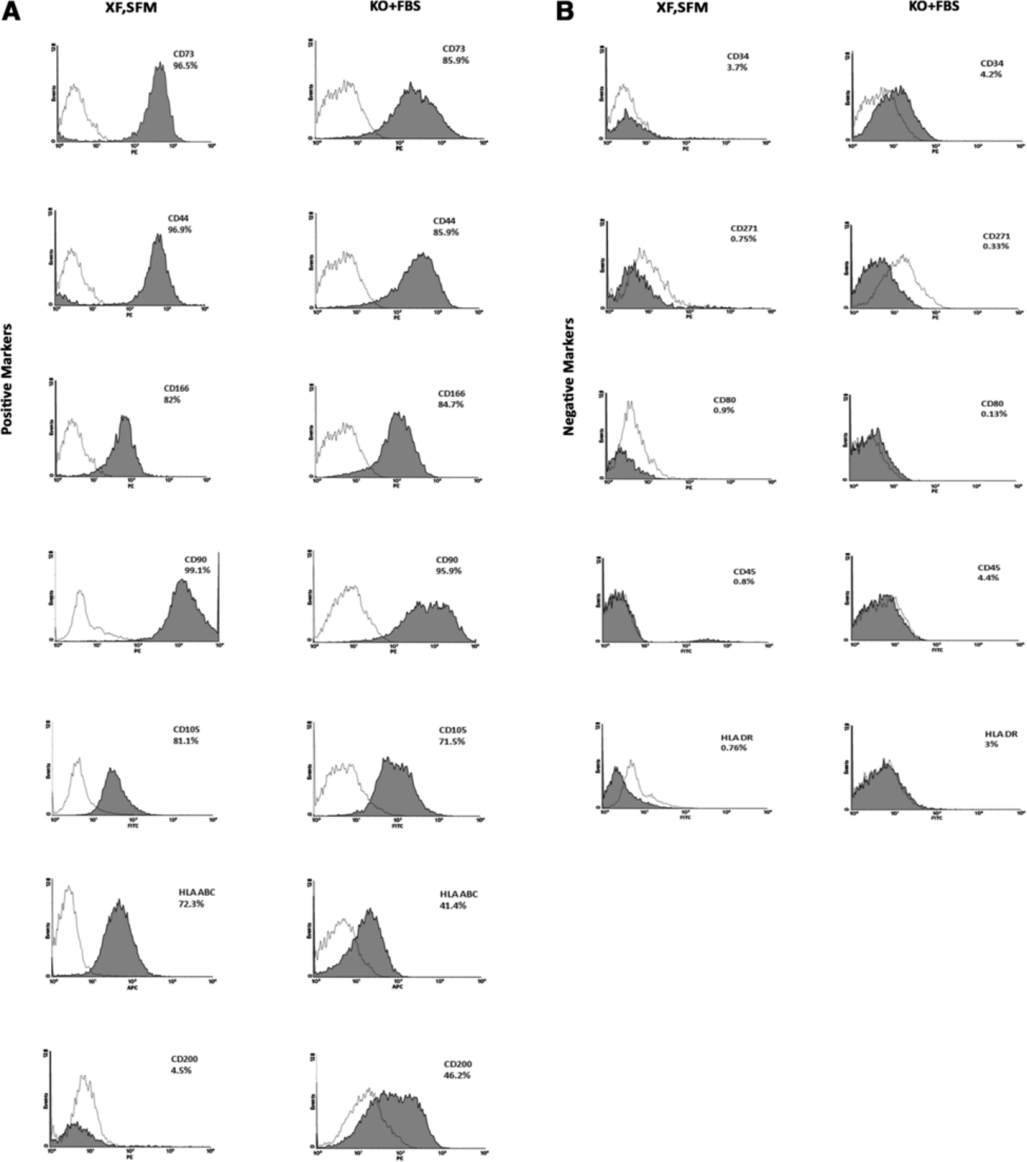
**Surface marker expression. A)** Histograms showing percentage expression of MSC-positive markers; **B)** Histograms showing percentage expression of MSC-negative markers. MSC, mesenchymal stem cells.

The immunophenotypic profile was evaluated by examining the cell surface marker expression on the expanded WJ-MSCs in both serum-free and serum-containing media at passage 3 and passage 5. A comprehensive panel of positive and negative CD markers was used to characterize the cells expanded in both conditions (Table 1). The cells expanded in serum-free and serum-containing conditions exhibited similar expression profiles for almost all the markers tested, which is in accordance with the data from previous studies [10], except for CD200, which is a member of the immunoglobulin super family and plays a role in immune regulation [16] (Figure 3, Table 3). Thus, the xeno-free serum-free medium used in this study did not seem to have altered any of the commonly analyzed MSC–associated markers when compared against standard conditions or culture duration.

**Table 3:** **Percentage expression of MSC-positive and negative markers, by cells cultured in MesenCult XFSM medium and in DMEM KO + 10% FBS medium, at passages 3 and 5**

KO + FBS	P3	P5	XF,SFM	P3	P5
CD markers	Average ± SD	Average ± SD	CD Markers	Average ± SD	Average ± SD
CD 73	93.4 ± 1.7	85.9 ± 15.7	CD 73	94.3 ± 4.09	96.5 ± 0.68
CD 34	5.1 ± 2.3	4.2 ± 3.2	CD 34	3.3 ± 2.5	3.7 ± 1.04
CD 44	92.6 ± 1.4	85.9 ± 15.8	CD 44	95 ± 3	96.9 ± 2.7
CD 166	81.6 ± 8.2	84.7 ± 0.4	CD 166	82.2 ± 11.09	82 ± 2.7
CD 90	98.5 ± 0.6	95.9 ± 1.9	CD 90	98.2 ± 0.6	99.1 ± 0.7
CD 200	52 ± 7.5	46.2 ± 8.8	CD 200	13.5 ± 12.3	4.5 ± 5.6
CD 271	0.6 ± 0.2	0.33 ± 0.2	CD 271	2.2 ± 1.8	0.75 ± 0.2
CD 105	85.5 ± 5.5	71.5 ± 17.0	CD 105	97.7 ± 1.9	81.1 ± 7.2
HLA DR	0.7 ± 0.7	3 ± 2.1	HLA DR	2.9 ± 2.5	0.76 ± 0.73
CD 45	0.4 ± 0.5	4.4 ± 3.1	CD 45	0.23 ± 0.2	0.8 ± 0.7
CD 80	2.7 ± 0.4	0.13 ± 0.05	CD 80	2.6 ± 2.9	0.9 ± 0.8
HLA ABC	73 ± 16.9	41.4 ± 8.6	HLA ABC	81.06 ± 1.8	72.3 ± 23.6

MSC, mesenchymal stem cell; P, passage; SD, standard deviation.

### Multilineage differentiation capacity

**Figure 4:**
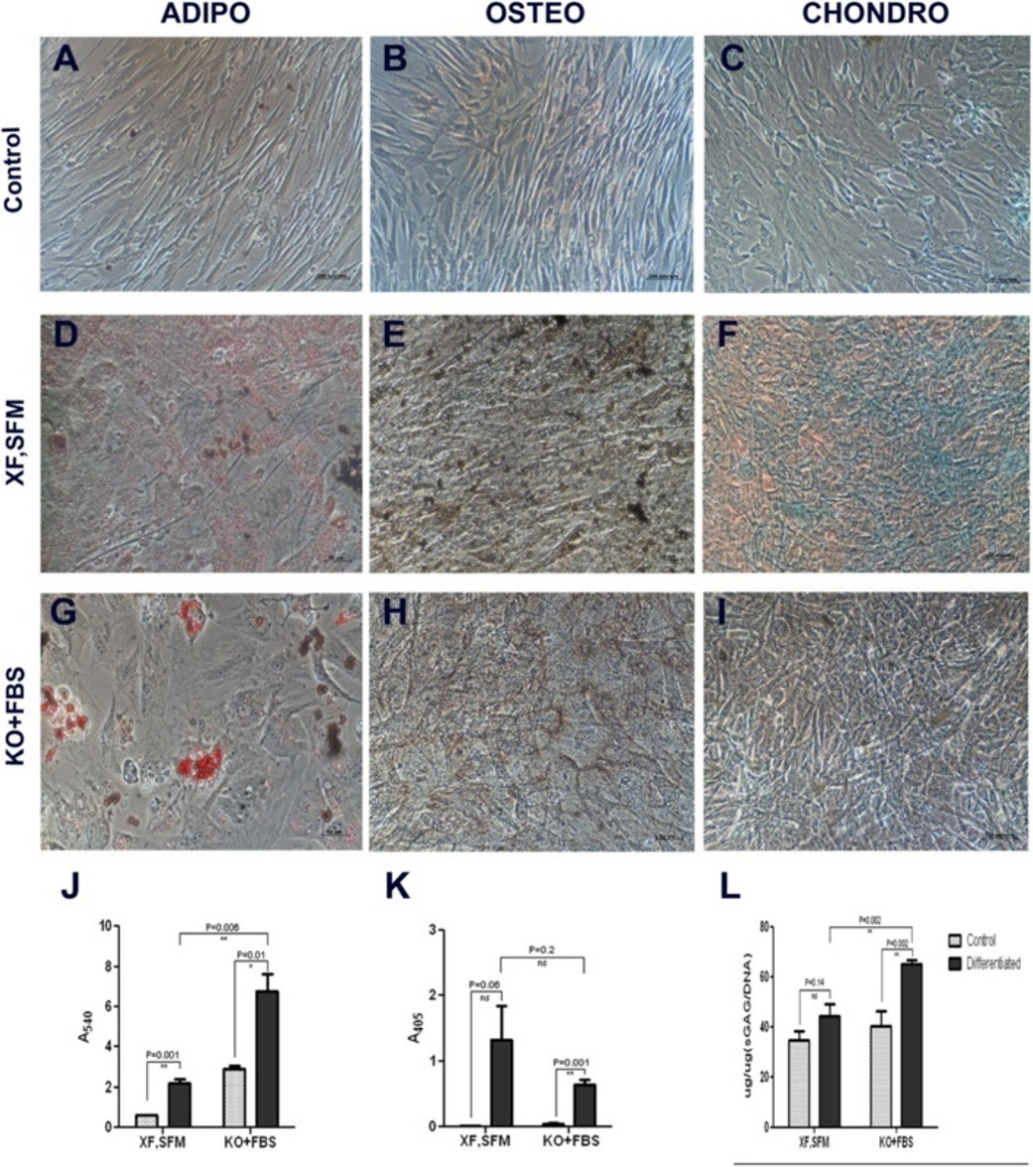
**Tri-lineage differentiation. A-I)** Representative images of XF,SFM-cultured and DMEM KO + 10% FBS-cultured MSCs, differentiated into adipocytes, osteocytes and chondrocytes, as visualized by Oil Red O, von Kossa and alcian blue stains, respectively, against undifferentiated controls; **J)** Bar graph to quantify oil droplets, post Oil Red O staining; **K)** Bar graph to quantify mineralization, post alizarin red staining; **L)** Bar graph to quantify sulfated glycosaminoglycan production by differentiated chondrocytes, normalized to DNA content. FBS, fetal bovine serum; MSCs, mesenchymal stem cells.

The multilineage differentiation capacity was evaluated for WJ-MSCs expanded under both conditions along adipogenic, osteogenic and chondrogenic lineages at passage 5 (Figure 4A-I). Adipogenic differentiation of the cells was detected by staining of accumulated lipid vesicles using Oil Red O stain. Uptake of the stain was then quantified by spectrophotometric analysis. The data show that the fold change above the undifferentiated control was similar for the cells expanded under both conditions. The cells expanded in Mesencult showed higher matrix mineralization following osteo differentiation as confirmed by von Kossa staining. Similarly, chondrocyte differentiation was estimated by positive staining of sulfated glycosaminoglycan (sGAG) after differentiation. Cellular sGAG content was quantified and subsequently normalized to the DNA content. Qualitative analysis did not reveal any significant difference in the differentiation capacity of WJ-MSCs expanded under both conditions, except for osteogenic differentiation, where XF, SFM cells showed increased *in vitro* osteogenic potential (Figure 4J,K and L).

### Immunogenicity and immunosuppressive capacity of WJ-MSC

**Figure 5:**
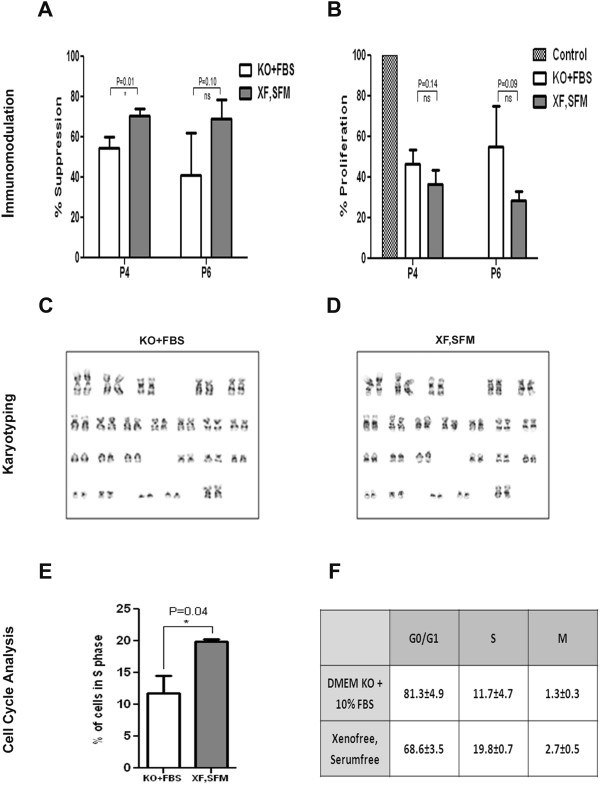
**Immunomodulation, karyotype and cell cycle analysis. A)** Bar graph showing percentage suppression of PBMC-generated MLRs, by cells cultured in XF,SF medium, against those cultured in DMEM KO + 10% FBS, at passages 4 and 6; **B)** Percentage proliferation brought about by co-culturing MSCs (cultured in both conditions) with human peripheral blood-derived PBMCs, at passages 4 and 6, shown against a one-way mixed lymphocyte reaction taken as 100% proliferation; **C and D)** Representative karyotypes of cells cultured in XF,SF medium and DMEM KO + 10% FBS, respectively, at passage 5; **E)** Percentage of cells cultured in XF,SFM and DMEM KO + 10% FBS, in S phase at passage 5; **F)** Percentage of cells cultured in XF,SF medium and DMEM KO + 10% FBS in G0/G1, S and M phases, at passage 5. FBS, fetal bovine serum; PBMC, peripheral blood mononuclear cells.

The immunogenicity of WJ-MSC grown in XF-SF- and FBS-containing media was tested in a one-way MLR assay against allogeneic PBMCs and the results are presented in Figure 5A. Compared to allogeneic PBMCs, both types of WJ-MSC population were significantly less immunogenic at both passages 4 and 6. We then compared the data between the two culture conditions. The cells expanded in XF-SF medium were less immunogenic compared to the cells grown in regular FBS medium; however, the difference was not significant (*P* = 0.09).

Immunosuppressive capacity of the cells cultured in both conditions was tested by comparing the ability to suppress a two-way MLR. Lymphocyte proliferation was strongly suppressed in the presence of WJ-MSCs isolated and expanded in both conditions (Figure 5B). The cells isolated and expanded in xeno-free medium were found to have better immunosuppressive capacity when compared to the standard FBS culture condition, although it was not found to be significant (*P* = 0.0621).

### Angiogenic potency of WJ-MSC

**Figure 6:**
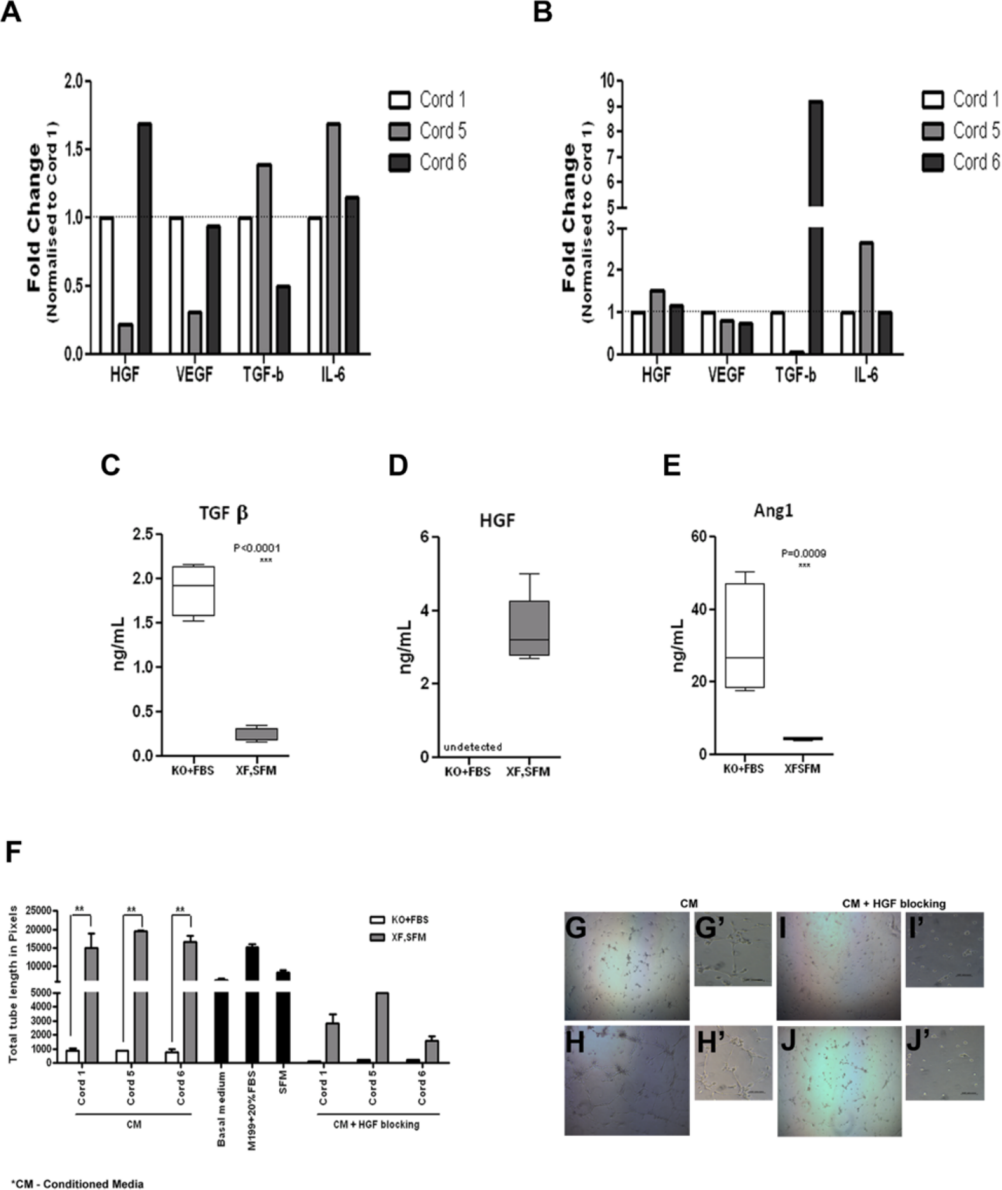
**Angiogenic potency. A, B)** Real-time PCR quantification of HGF, VEGF, TGFβ-1 and IL6 for cells cultured in DMEM KO + 10% FBS and in XF,SFM, all normalized against Cord 1 (DMEM KO + 10% FBS); **C,D,E)** Box graphs representing variations observed between cells cultured in XF,SFM and FBS-containing medium, when analyzed for cytokine secretions of TGFβ, HGF and Ang1; **F)** Tube formation efficiency (estimated using HUVEC, cultured on growth factor reduced Matrigel) of WJ-MSC-conditioned medium, from three cords cultured in DMEM KO + 10% FBS and in XF, SFM. Anti-HGF neutralizing antibody was used to block tube formation in each condition. **G-J’)** Representative 4 X pictures showing tubes formed. FBS, fetal bovine serum; HGF, hepatocyte growth factor; TGFβ1, transforming growth factor-β1; VEGF, vascular endothelial growth factor. ** signifies extent of significance of the numeric value.

In order to evaluate the angiogenic potency, we characterized the secretome for angiogenic cytokines in the conditioned medium (CM) collected from xeno-free and serum-containing conditions. The levels of VEGF, Ang1, HGF and TGF-beta1 were estimated by ELISA. The levels of these cytokines in individual samples are provided in Table 4. VEGF was completely absent in both culture conditions whereas a measurable difference was noticed in the secretion profile for the other cytokines between the two conditions. The secretion profile was uniform in the XF, SFM in comparison to the cells grown in the serum-containing medium (Figure 6C, D and E). CM from XF, SFM established cultures had uniform secretion of the angiogenic proteins and it correlated with gene expression data (Figure 6A and B). Further, we confirmed the functional efficacy by the Matrigel tube formation assay using HUVEC cells. HUVEC cells were plated on to the growth factor reduced Matrigel with CM collected from the two conditions. CM from both conditions was able to promote tube like structures within six hours. CM from XF, SFM promoted longer tube structures and branch points in comparison with serum-containing CM (Figure 6G-J’, Additional file 3: Figure S3). As the cytokine analysis data revealed that the HGF is significantly higher in XF, SFM CM, we validated the role of HGF in an *in vitro* tube formation assay using blocking anti HGF antibody. Upon blocking HGF, we observed a significant reduction in the tube length and branch points at six hours. Through these results, we have clearly established that HGF is the main factor responsible for the *in vitro* angiogenic properties contained within the CM from XF, SFM (Figure 6F and G-J’).

**Table 4:** **Expression profile of pro-angiogenic cytokines, as observed in the secretome of cells cultured in MesenCult XF,SFM and KO + FBS media**

		XF,SFM (ng/ml)			KO + FBS (ng/ml)	
Cytokines	Cord 1	Cord 5	Cord 6	Cord 1	Cord 5	Cord 6
HGF	3.15 ± 0.49	2.8 ± 0.14	4.5 ± 0.7	40 ± 0.2	ND	ND
TGF-β-1	0.24 ± 0.08	0.17 ± 0.01	0.27 ± 0.1	2.14 ± 0.02	1.9 ± 0.1	1.5 ± 0.07
Ang-1	3.85 ± 0.07	4.75 ± 0.07	4.62 ± 0.02	18.1 ± 0.5	48.25 ± 3.1	26.7 ± 2.1
VEGF	ND	ND	ND	ND	ND	ND

### Genomic stability, senescence and transformation marker status and cell cycle analysis of expanded WJ-MSC in xenofree culture

To determine the impact of large scale expansion of the WJ-MSCs on the genomic stability, we subjected the cells expanded in both conditions to karyotype analysis on metaphasic WJ-MSCs at passage 5. The analysis revealed no gross chromosomal aberrations, thus suggesting that the cells preserved their normal karyotype in both conditions (Figure 5C and D).

**Figure 7:**
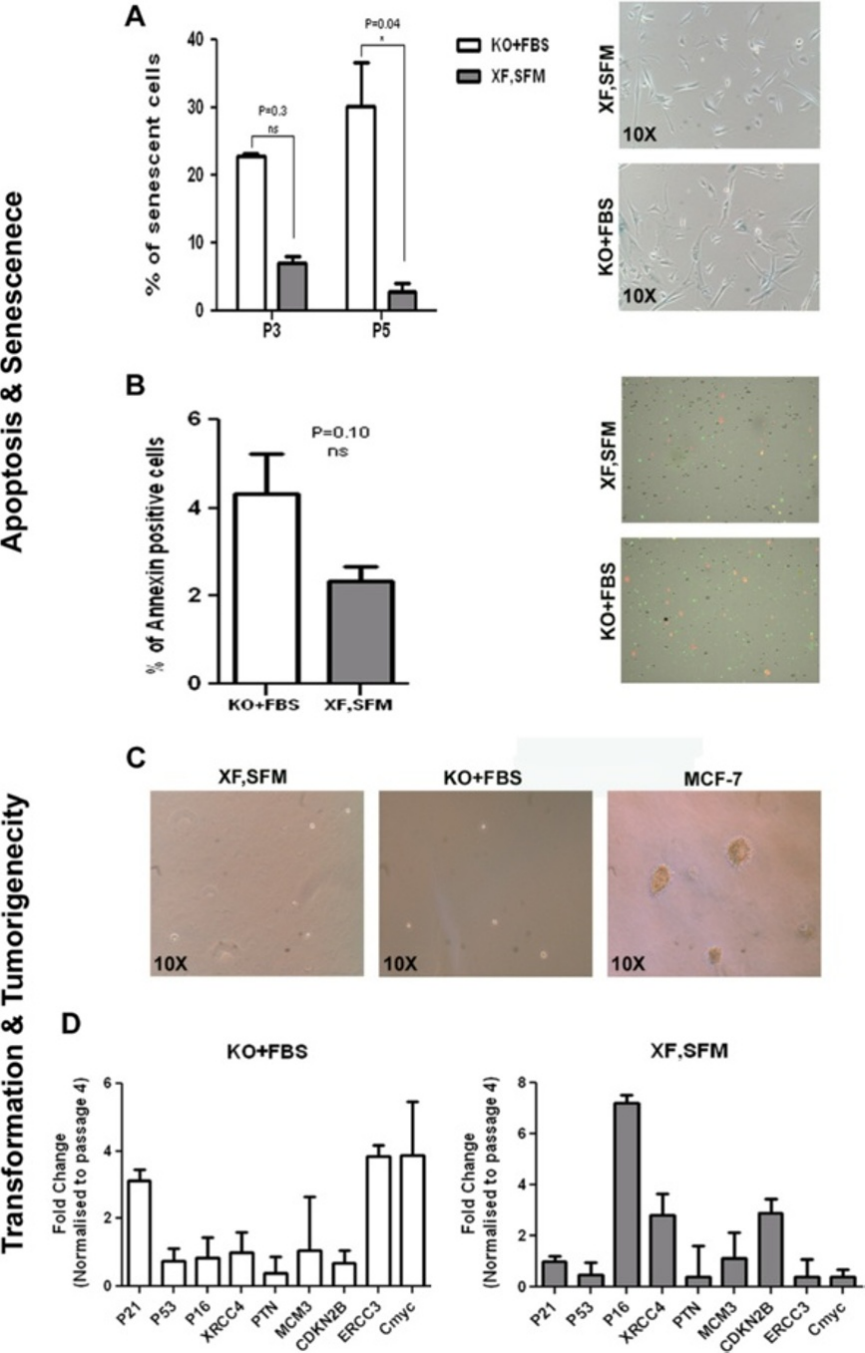
**Senescence, apoptosis and transformation. A)** Percentage of senescent cells in XF,SFM cultures, compared against DMEM KO + 10% FBS cultures at P5 followed by representative 10 X images of fields showing blue colored senescent cells; **B)** Percentage of Annexin positive (apoptotic) cells in XF,SFM cultures compared to DMEM KO + 10% FBS cultures at P5 followed by representative images of fields showing green colored apoptotic cells; **C)** Representative pictures of cells cultured in soft agar medium, showing no colonies in XF,SFM and DMEM KO + 10% FBS cultures, compared to colonies observed in MCF-7 cultures; **D)** Comparative gene expression analysis between cells cultured in XF,SF medium and DMEM KO + 10% FBS at P6. FBS, fetal bovine serum; P, passage.

MSCs are also known to undergo replicative senescence *in vitro* on extended passaging, which is characterized by decreased proliferation, cell size enlargement, replicative quiescence and an increase in senescence associated-β-gal activity. We employed the enzyme lysosomal pH 6.0 β-galactosidase as a senescence marker to obtain an estimate of the percentage of senescent cells at passage 3 and passage 5, using serum-free and xeno-free media. We observed considerable differences between the WJ-MSCs grown in XF, SFM versus the cells that were expanded in serum-containing medium at both passage 3 and passage 5 (Figure 7A). While the percentage of senescent cells in XF, SFM remained 7.12 ± 2.82 (passage 3) and 1.53 ± 2.11 (passage 5), in serum-containing medium the percentage of senescent cells progressively increased from 22.79 ± 0.77 (P3) to 30.22 ± 11.07 (passage 5) (Figure 7A). We also investigated the apoptotic status of cells by Annexin V staining. The cells cultured in XF, SFM showed a lower number of apoptotic cells when compared to cells expanded in the standard condition, although this difference was not significant (*P* <0.12) (Figure 7B).

MSCs are known to adapt or acquire chromosomal aberrations after extended passaging [17]. In order to ensure the safety of WJ-MSCs expanded in XF, SFM for clinical use, we extensively characterized the cells and subsequently looked at the *in vitro* tumorigenic potential of these cells. Tumorigenic potential was assessed by soft agar assay for colony formation to determine the transformation status of the expanded cells. The cells were cultured for almost a month and following the incubation period the number of colonies formed per well was quantified. The cells isolated and expanded in both conditions failed to form any colonies even after one month of incubation. The human breast cancer cell line MCF-7 was used as the positive control and large colonies were observed at the same seeding density (Figure 7C). We also compared the gene expression of transformation- and senescence-associated markers, such as P21, P53, P16, XRCC4, ERCC3, PTN, MCM3, CDKN28 and c-myc expression by RT-PCR of the cells cultured under both conditions. The expression of P53, XRCC4, PTN, CDKN2B and MCM3 was similar in cells cultured under both conditions whereas with respect to P21, P16, ERCC3 and cmyc we observed a difference in the expression levels which was not significant (Figure 7D). These results clearly suggest that the scaled up WJ-MSCs in the XF, SFM condition did not exhibit potential for transformation. Since the WJ-MSCs proliferated rapidly under the XF, SFM condition, we examined the cell cycle status of these cells. For identification of cell distribution during the various phases of the cell cycle, we performed flow cytometry analysis by staining the cells with PI. The cells cultured in Mesencult had 19.8 ± 0.7% cells in S phase whereas for the KO + FBS cultures the value was 11.7 ± 4.7%. Similarly, for the G0/G1 phase the percentage of cells in serum-free and serum-containing media was 68.6 ± 3.5 and 81.3 ± 4.9, respectively. In the G2 + M phase the percentages were 1.3 ± 0.3 and 2.7 ± 0.5 for DMEM KO + FBS and XF, SFM cultures, respectively. These results indicate that cells cultured in XF-SF medium possessed higher proliferation capacity with the largest proportion of cells found within the S phase (Figure 5D and E).

## Discussion

WJ-MSCs have gained significant attention in recent years because of their similarity with MSCs obtained from other sources as well as due to their ease of isolation and *ex-vivo* expansion. Moreover, these cells display a majority of the properties of MSCs, such as multipotency, higher proliferation rates, immunomodulatory properties and secretion of paracrine factors [18]. As a result, WJ-MSCs are believed to be potentially ideal candidates for clinical applications for various disease indications. However, in order to use these cells in clinical settings, it is mandatory to establish a consistently reproducible large scale production process [19, 20] and to define a standard operating procedure for a good manufacturing practice (GMP)-compliant production of MSCs [21], including selection of an optimal cell therapy-compliant culture medium. We had earlier reported that WJ-MSCs could be expanded to clinical scale in reasonable time without altering their basic characteristics [10]. Here we have extended our findings to report a completely xeno-free serum-free isolation and expansion process for the clinical scale expansion of WJ-MSCs. Most current isolation and expansion procedures involve the use of FBS as the main ingredient which does not have a defined number of components and presents the risk of transmission of unknown infectious agents. Thus, it has become evident that culturing of MSCs in defined medium that is FBS-free for large scale expansion of these cells is critical for translational research [22]. In this study, we tested two commercially available xeno-free and serum-free media for the isolation and large scale expansion of WJ-MSC. Of the two media tested, Mesencult XF, SFM was found to be best suited for the isolation and expansion of WJ-MSC to a clinical scale in our hands. Xeno-free serum-free established cultures of WJ-MSCs were extensively characterized and compared with the cells expanded using a standard FBS-containing medium. We have adapted the explant culture technique instead of the collagenase digestion method which was reported earlier [23]. In addition, instead of porcine-derived trypsin, a cell dissociation enzyme, TrypLE Select, free of any animal derived component, was used for dissociation of cultured MSCs. Several groups have reported on the isolation of MSCs from various tissue sources in xeno-free, serum-free conditions in the recent past [24]. This is the first report to demonstrate the isolation and large scale expansion of WJ-MSCs in completely xeno-free and serum-free conditions.

WJ-MSCs expanded in xeno-free, serum-free conditions had significantly higher yield compared to FBS containing medium. We had earlier optimized a protocol for scale-up to 10^8^ WJ-MSCs from a single umbilical cord within a time period of 15 days [10]. Using the current xeno-free, serum-free culture process, it took us approximately 13 days whereas for the DMEM KO +10% FBS, it took 15 days to arrive at the specified cell number.

In order to meet with clinical translational requirements, we further analyzed the post-freeze thaw viability and characteristics of the cells cultured in both conditions. MesenCult XF, SFM cultured cells were frozen in a commercially available serum-free cryopreservation solution, containing 10% dimethylsulfoxide (DMSO), while DMEM KO + FBS cultured cells were frozen in the traditional FBS + 10% DMSO mix. Upon thawing the cells frozen at passage 5, we observed that the cell viability was between 92% and 100% for both conditions (data not shown).

Furthermore, culturing the cells under the XF-SF condition did not result in significant variation of their typical MSC marker expression profile. However, we noticed changes in the expression of CD200 levels between the two culture conditions used. Generally, MSCs expressing CD200 are known to suppress TNF α production by CD200 receptor positive macrophages. Among the different MSC sources, WJ-MSCs express CD200 in a higher proportion and they have been reported to play an important role in regulating immune responses [16]. Cells expanded in xeno-free and serum-free medium contain a lower percentage of CD200 positive cells. Although the expanded cells expressed a lower percentage of CD200 positive cells, these WJ-MSCs exhibited higher immunosuppression activity in the MLR assay when compared to the cells expanded in FBS-containing media. Since immunosuppression activity is a multifactorial process, the exact mechanism through which the Mesencult grown cells render immunosuppression remains to be determined. The cells cultured under both conditions were negative for HLA-DR and the co-stimulatory molecules CD40, CD80 and CD86, which could potentially induce T cell anergy tolerance and explain the immune privileged status of MSCs [25, 26]. In addition, there are several other factors that have been proposed to play a critical role in regulating the immunosuppressive effects of MSCs, including HLA-G, nitric oxide, indoleamine 2,3-dioxygenase (IDO), HGF, TGFβ and so on.

Multilineage differentiation potential, which is the hallmark of these MSCs, was not compromised by the xeno-free, serum-free culture conditions. It has been reported that WJ-MSCs have lower adipo and chondro differentiation potential when compared to bone marrow and other sources of MSCs. We observed that the xeno-free and serum-free cultures have lower adipo and more osteo and chondro differentiation potential when compared with serum-containing cultures. Our results corroborated the earlier report which has demonstrated robust osteogenic differentiation with the xeno-free and serum-free cultures [15]. Clonal expansion property as estimated by the CFU-F assay unequivocally demonstrated the superiority of the cells expanded under xeno-free, serum-free conditions.

The proposed mechanisms of action of MSCs are believed to occur primarily through their paracrine activity [27]. We observed that the cells grown under XF-SF conditions displayed a more consistent pattern of angiogenic factors secretion compared to the FBS grown cells. The secretion profile also correlated with the gene expression data shown in Figure 6. When the functional activity of the angiogenic factors was assessed *in vitro*, we observed that the CM derived from XF SF cultures showed increased neo angiogenesis. These results corroborated well with earlier reports published by Corotchi *et al*. and Choi *et al*., using WJ-MSCs [15, 28].

It is known that long-term culture of primary cells might also have therapeutic consequences, although this has not been adequately addressed in the ongoing trials [21]. Furthermore, there is a growing perception that even under highly standardized culture conditions, the probability that continuous culturing of cells may eventually lead to replicative senescence is a hypothesis that further warrants experimental investigation [29, 30]. Although there are no specific molecular markers to track the cellular aging in MSCs, we have addressed the replicative senescence issue by examining senescence associated-β-gal activity in our cell populations. In the XF, SFM condition we were not able to detect a significant number of senescent WJ-MSCs, and similarly very few apoptotic cells were observed in our cultures. To further analyze the senescence-associated marker profile in these cells we measured the expression of several cell cycle associated genes. For the most part, the expression profile was similar between the two cell types except for a few genes whose function has been attributed to cell proliferation and DNA repair [29]. Proliferation under non-physiologic *in vitro* culture conditions could result in mutations and chromosomal aberrations. Conventional karyotype analysis of the cells in both conditions has not revealed any major genomic gains or losses and we did not observe cells with transforming potential in our culture conditions.

Based on the results reported here, Mesencult XF, SFM appears to be a suitable medium of choice for the large scale expansion of WJ-MSCs, without compromising the quality of these cells. We observed that Mesencult medium supported the growth and expansion of WJ-MSCs that could be taken forward for clinical application. Alternatives to FBS, such as human AB serum or platelet lysates, cannot be considered as a better choice for producing MSCs for clinical use since the regular availability of consistent reagents may be difficult. Although the successful isolation of MSCs has been performed with serum and in the absence of it, for most serum-free, xeno-free commercially available solutions, effective cell isolation remains challenging [31]. Initial cell adhesion in xeno-free and serum-free cultures still remains a major problem since most commercial serum-free media require precoating of culture vessels, which makes it difficult for large scale expansion in cell stacks. However, in this study we have successfully demonstrated the feasibility and optimization of isolation and large scale expansion of WJ-MSC more efficiently and consistently, in the large numbers required to meet the clinical demand. Based on the data presented in this report, we believe that these cells could be therapeutically effective in preclinical and clinical evaluation.

## Conclusions

In summary, safety is the first objective to meet in cellular therapy and this warrants the production of safe stem cell products and requires extensive quality monitoring to ensure that the cells maintain overall phenotype and functional potential and exhibit normal genotype. We have systematically developed a large scale production process, using completely xeno-free, serum-free medium. Extensive characterization revealed that the WJ-MSCs that are expanded in Mesencult xeno-free, serum-free medium consistently exhibit greater angiogenic potential when compared to those expanded under standard serum containing conditions.

## Electronic supplementary material

Additional file 1: Figure S1: A – E - Morphology pictures of WJ derived MSCs cultured in MesenCult XF,SM Media, from P0 to P7; F and G - Cumulative population doubling and total cell number of cells cultured in MesenCult XF,SF Media, from passage 0 to passage 5. (TIFF 1 MB)

Additional file 2: Figure S2: A, B and C - Cumulative population doubling, population doubling time and population doublings of cells cultured in MesenCult XF,SM Medium, versus cells cultured in DMEM KO + 10% FBS. (TIFF 964 KB)

Additional file 3: Figure S3: Tube forming efficiency - total number of branch points observed between cells cultured in serum-free and serum-containing media. (TIFF 2 MB)

## Abbreviations

DMEM KO: (Dulbecco’s) modified Eagle’s medium
BM: bone marrow
bp: base pair
BrdU: bromodeoxyuridine
CFU-F: colony forming unit-fibroblast
CM: conditioned medium
CPD: cumulative population doubling
DPBS: Dulbecco’s phosphate buffered saline
EDTA: ethylenediaminetetraacetic acid
ELISA: enzyme-linked immunosorbent assay
ERCC3: excision repair cross-complementing rodent repair deficiency complementation group – 3
FBS: fetal bovine serum
GvHD: graft versus host disease
HGF: hepatocyte growth factor
HUVEC: human umbilical vein endothelial cells
ICSCRT: International Committee for Stem Cell Research and Therapy
IEC: Institutional Ethics Committee
IL6: interleukin 6
MCF-7: Michigan Cancer Foundation-7
MCM3: minichromosome maintenance complex component 3
MLR: mixed lymphocyte reaction
MSCs: mesenchymal stem cells
P16: cyclin-dependent kinase inhibitor 2A
P21: cyclin-dependent kinase inhibitor 1
P53: tumor protein 53
PBMC: peripheral blood mononuclear cells
PD: population doubling
PI: propidium iodide
PTN: pleiotrophin
RPMI 1640: Roswell Park Memorial Institute 1640 medium
RT-PCR: reverse transcriptase-polymerase chain reaction
SD: standard deviation
sGAG: sulfated glycosaminoglycan
TGF: transforming growth factor
TNF: tumor necrosis factor
VEGF: vascular endothelial growth factor
WJ: Wharton’s jelly
XF: SF, xeno-free, serum-free
XRCC4: X-ray repair cross-complementing protein 4.
