# Supplementary figures and images for: Are serum-free and xeno-free culture conditions ideal for large scale clinical grade expansion of Wharton’s jelly derived mesenchymal stem cells? A comparative study
Source: Stem Cell Res Ther. 2014 Jul 28;5(4):88. doi: 10.1186/scrt477 (PMC4247668; doi:10.1186/scrt477)

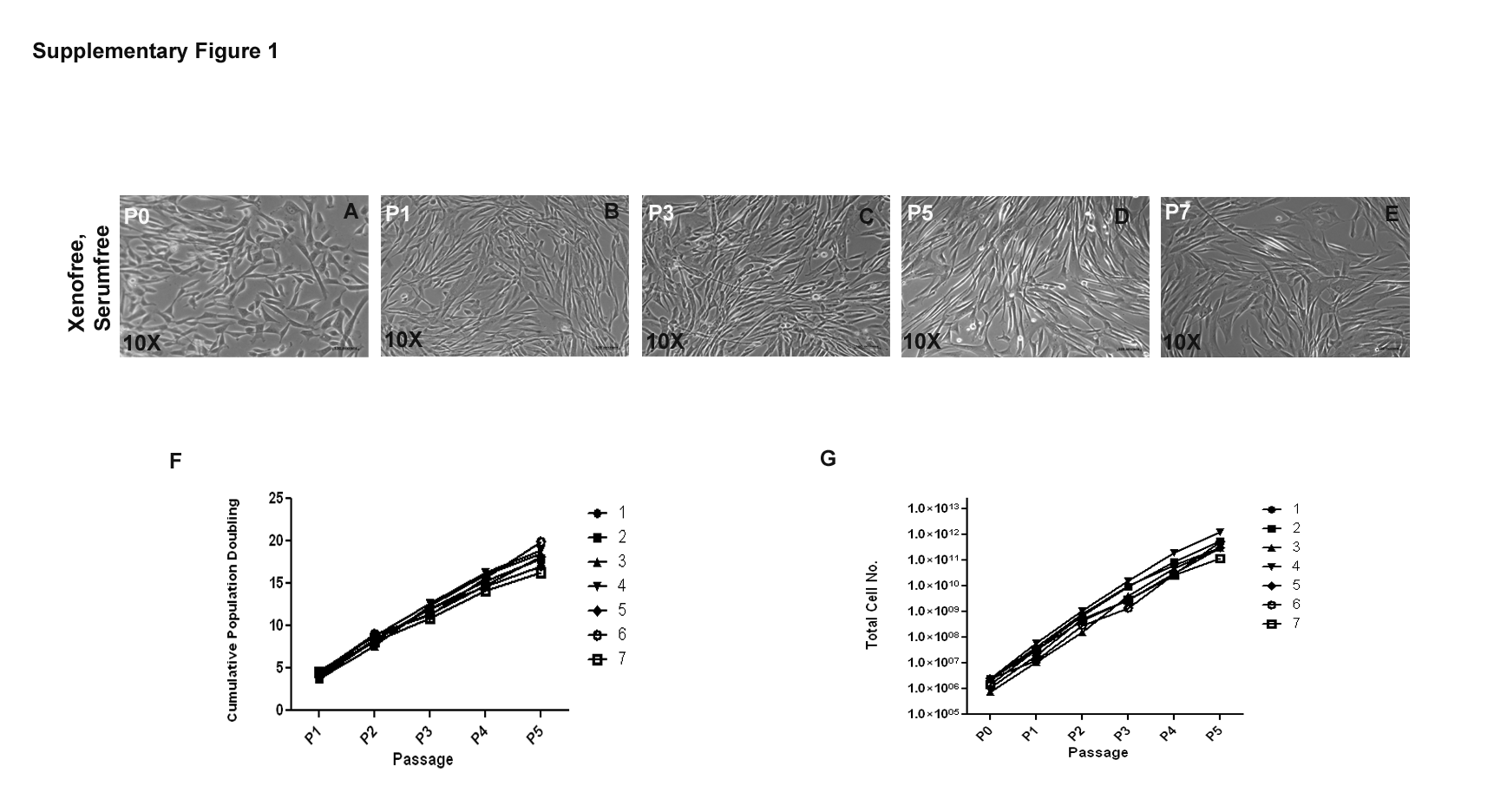

Supplement: Supplementary file 1 — Additional file 1: Figure S1: A – E - Morphology pictures of WJ derived MSCs cultured in MesenCult XF,SM Media, from P0 to P7; F and G - Cumulative population doubling and total cell number of cells cultured in MesenCult XF,SF Media, from passage 0 to passage 5. (TIFF 1 MB) [file 13287_2013_401_MOESM1_ESM.tiff]

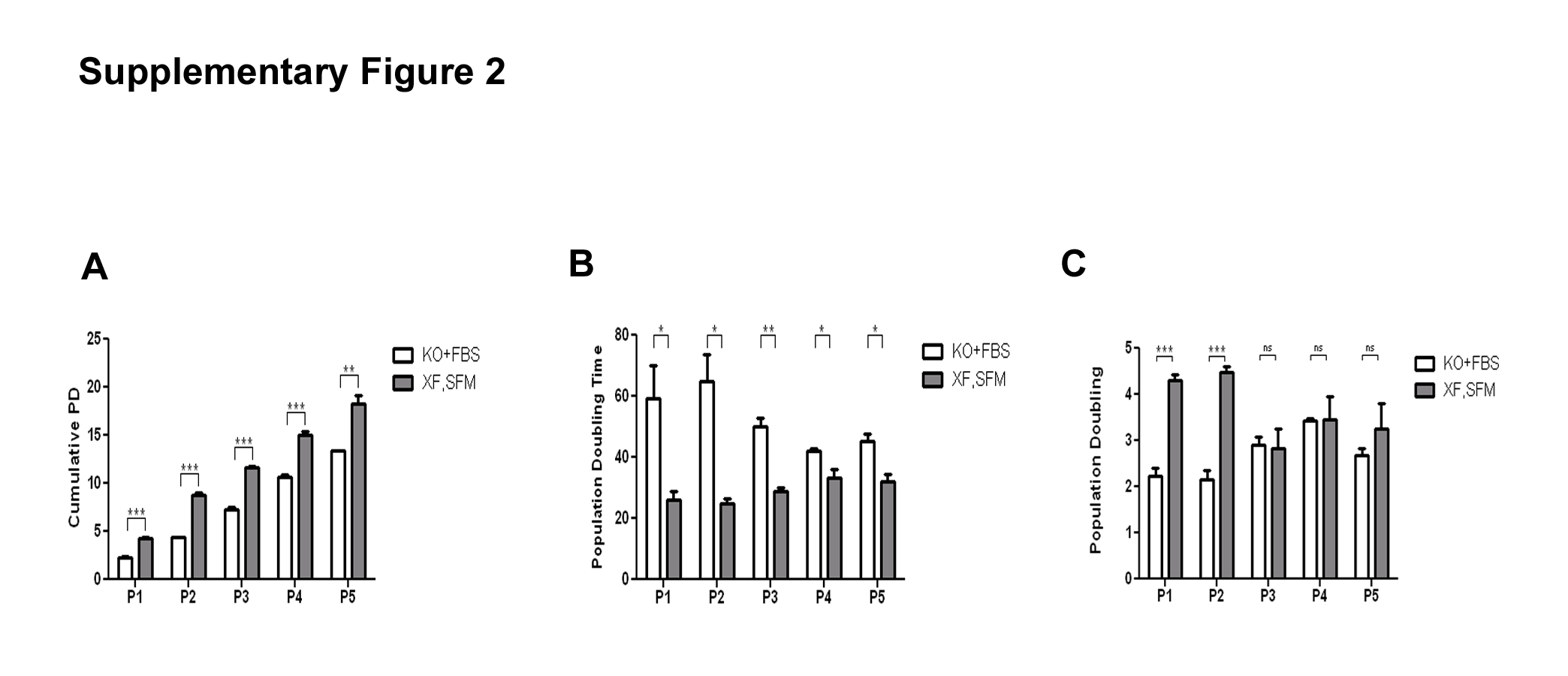

Supplement: Supplementary file 2 — Additional file 2: Figure S2: A, B and C - Cumulative population doubling, population doubling time and population doublings of cells cultured in MesenCult XF,SM Medium, versus cells cultured in DMEM KO + 10% FBS. (TIFF 964 KB) [file 13287_2013_401_MOESM2_ESM.tiff]

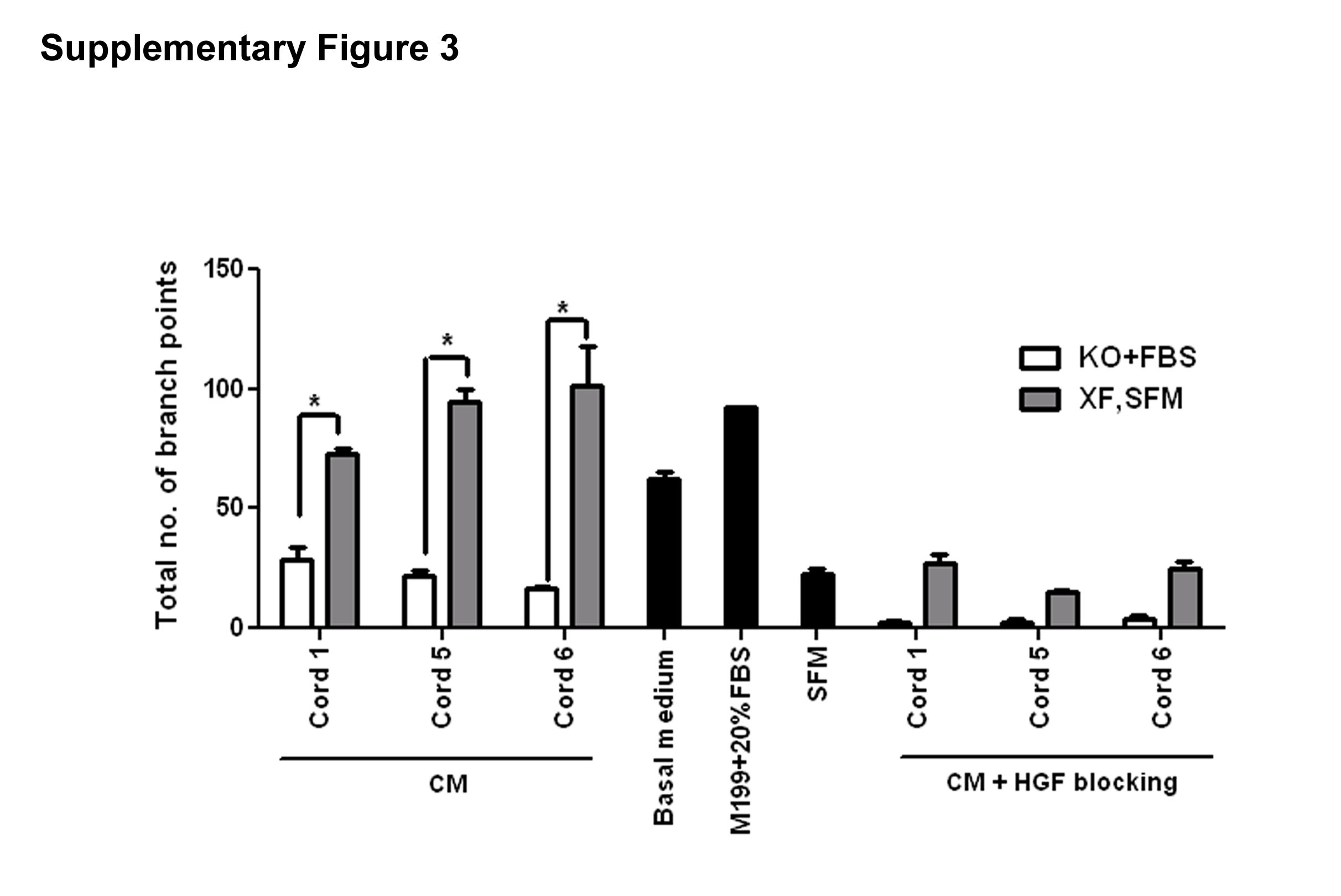

Supplement: Supplementary file 3 — Additional file 3: Figure S3: Tube forming efficiency - total number of branch points observed between cells cultured in serum-free and serum-containing media. (TIFF 2 MB) [file 13287_2013_401_MOESM3_ESM.tiff]
